# An Essay on the Use of Holly and Ilicine in the Cure of Intermittent Fevers,(for Which the Silver Medal of the Session 1832 Was Awarded.)

**Published:** 1832-08

**Authors:** L. F. E. Rousseau

**Affiliations:** Chef des Travaux Anatomiques au Muséum d'Histoire Naturelle de Paris, Membre de plusieurs Sociétés savantes, nationales et étrangères, &c.


THE LONDON

### Medical and Physical Journal.

402, VOL. LXVIII.] AUGUST 1832. [74, *New Series.*

"For many fortunate discoveries in medicine, and for the detection of numerous errors, the world is indebted to the rapid circulation of Monthly Journals; and there never existed any work to which the Faculty, in Europe and America, were under deeper obligations than to the 'Medical and Physical Journal of London,' now forming a long but invaluable series."—RUSH.

### ORIGINAL PAPERS.

TRANSACTIONS OF THE MEDICO-BOTANICAL SOCIETY OF LONDON,

*Published under the Direction of the President and Council.*

### HOLLY AND ILICINE.

AN ESSAY ON THE USE OF HOLLY AND ILICINE IN THE CURE OF INTERMITTENT FEVERS, (*for which the Silver Medal of the Session* 1832 *was awarded.*) By L. F. E. ROUSSEAU, M.D., Chef des Travaux Anatomiques au Muséum d'Histoire Naturelle de Paris, Membre de plusieurs Sociétés savantes, nationales et étrangères, &c.

(Read at a general Meeting of the Fellows, 12th June, 1832; Earl STANHOPE, President, in the chair. Translated and condensed by GEORGE CLENDINNING, Esq., Secretary.)

THE Medico-Botanical Society of London having proposed to award a silver medal to whomsoever should make known "the medicinal qualities and uses of any indigenous plants the properties of which are not yet sufficiently acknowledged, or any new uses and applications of other indigenous plants which may have been unduly neglected or forgotten," I purpose presenting for the *concours* proposed by that very learned Society the accompanying paper, exemplifying the great advantages I have reaped from the use of the leaves of the common holly (Ilex Aquifolium) in the treatment of intermittent fever, &c.

The unjust neglect which this indigenous shrub has experienced by its omission in the modern materiæ medicæ has for many years engaged my attention. I have also solicited the co-operation of many of my colleagues, whose conclusive observations bear testimony to the facts which I have the honour to present, in order to obtain a recompence which it is more easy anxiously to desire than actually to gain.

### *Of the Virtue of the Leaves of the Holly (Ilex Aquifolium) in the Treatment of Intermittent Fever.*

"Gratia sic minimo magna labore venit."

I am indebted to numerous experiments, and to their successful results, for the advantage of proposing the holly as a febrifuge and succedaneum for cinchona bark.

The experience of twenty years has convinced me of the virtue of this medicine, which, administered by enlightened and intelligent men, has fulfilled all the conditions required of it to supersede the Peruvian bark in the treatment of intermittent fever.

Although it is one alone that here principally concerns us, it may not be impertinent to state that there are at least twenty-five species of holly growing in the four divisions of our globe, which form a very numerous genus: however, we cannot assign to any of these the febrifuge properties which we have observed. In the indigenous holly, which grows in France and in all parts of Europe: for this reason, the species in question is the only one which has been submitted to experiment by me.

[The author here gives at some length the botanical and physical characters and history of the Ilex Aquifolium, which, as they differ not materially from accounts already published, and familiarly known to almost every one, it has been deemed advisable to omit these paragraphs in the translation, and to proceed at once to the]

*Chemical analysis.* We are indebted to M. Lassaigge, a zealous friend of science, for the first analysis which he undertook to discover the composition of the leaves of the holly. This analysis furnished him, viz. 1, wax; 2, chlorophylle, (green matter of the leaves;) 3, a bitter matter, which is neutral, non-crystallizable, soluble in alcohol, and undecomposable by the acids and alkalies; 4, a yellow colouring matter; 5, gum; 6, acetate of potass; 7, muriate of potass and lime; 8, supermalate of lime; 9, sulphate and phosphate of lime; 10, fibre of the wood (lignin).

The febrifuge principle appearing to me to reside in the bitter matter, it was necessary, in order to ascertain its presence, to have recourse to new processes. M. Déleschamps, an able chemist, has obtained the following results:

First process. A kilogramme of the leaves of the holly, previously dried and reduced to powder, was twice boiled, each time in five kilogrammes of water, during two hours, in a Papin's digester, to facilitate the dissolving of the bitter principle, by increasing the temperature. After the second boiling, the residue of the powder being deprived of all taste, the liquids were mixed, filtered, and evaporated to three fourths. On the addition of a solution of subacetate of potass, (which was added in excess,) the liquid assumed a thick viscous appearance. A solution of two ounces of subcarbonate of potass being then added, it appeared more limpid on being well stirred: this not only destroyed the viscosity which impeded the filtration, but threw down the subacetate of lead in excess. After having refiltered, half an ounce of dilute sulphuric acid was added, which caused a white precipitate. The liquid, being acid, was saturated with carbonate of lime, and, being again filtered, it was evaporated to the consistence of an extract, which possessed all the bitter taste of the plant. I treated this matter with alcohol of 40°, and decanted off the clear portion, and by evaporation obtained a brown substance, attracting moisture very rapidly, having a very bitter taste, and rather an agreeable odour. I dried this product, and procured it in shining laminæ. I then treated that portion of the extract which was not dissolved by alcohol at 40°, with alcohol at 36°, and obtained by the same means a product, which only differed from the first, by being of a deeper yellow colour.

Second process. The product of the decoction evaporated to the consistence of an extract, was treated with alcohol at 36°, and the spirit renewed until nothing further could be dissolved. The liquids were all then mixed and evaporated, and the product procured dried, and then put into water at the temperature of 40°. After half an hour, the liquid was filtered, and precipitated by a solution of subacetate of lead, the excess of which was thrown down by sulphuretted hydrogen. It was again filtered, and evaporated in a porcelain vessel to the consistence of an extract, which was treated with alcohol at the temperature of 36° (therm. Reaum.), and afterwards dried as in the first process.

This second process being more expensive, and less bitter principle being obtained than by the first, it is therefore not to be commended.

Third process. This consists in making an alcoholic extract of the leaves, which should be mixed with water, and afterwards treated with subacetate of lead, sulphuric acid, and carbonate of lime; and alcohol should then be added, and the product, when filtered, evaporated, and dried, gives a substance which presents the following characters, when submitted to the following different tests: The acids do not decompose it, unless the temperature be raised; it then assumes a black appearance, and disengages an empyreumatic odour. The alkalies do not act upon it; chlorine neither changes its colour, nor its chemical nor medical properties. It is precipitated neither by the nitrate of silver, hydrochlorate of platinum, acetate of potass, nor by the oxalates of potass and ammonia. It is insoluble in ether; soluble in alcohol at 40°, equally so in alcohol at 36°, and even in warm water.

This product, which I have named Ilicine, is of a brown colour; rapidly absorbs moisture, which probably renders it non-crystallizable: heated in a platinum crucible, the ilicine gives carbon, and betrays the presence of an alkali, by restoring the blue colour of litmus paper reddened by an acid: this alkali proceeds from a salt, having potass for its base.

*Results.*

2 lbs. of dried leaves give of dry extract, ℥v., gr. iv.
2 lbs. . . . . give of ilicine, ℥ij., gr. xlij.
2 lbs. . . . . lose by drying, 1 lb., ℥iv.
2 lbs. of fresh leaves give of dry extract, ℥iij. ʒiv. gr. xxiv.

*Medical Properties.*

Those authors who have spoken of the holly consider it as possessing emollient, resolving, diuretic, and expectorant properties: few consider it as a febrifuge. Before it was employed internally, many remarked that the bruised and putrified bark yielded a viscous substance called birdlime, and which was applied externally as a cataplasm, supposing it to have resolving or maturative qualities: with this idea, M. Chomel employed it to alleviate the pain of gout.

The root, according to many, has the same emollient and resolving properties as the bark. The decoction of the leaves, or their powder, has been praised in catarrh, pleurisy, colic, and the diseases of the urinary organs. The great Haller has recommended the juice of the leaves in icterus; and we shall perceive, in many of the observations which follow, that the alvine evacuations which were produced in some patients depended on its action on the liver, as Haller suspected when recommending it in jaundice.

I remarked, that few physicians have regarded it as a febrifuge, which admits of a simple explanation; as Durande and Reil are the only persons who have sought after a new therapeutical remedy for fevers. Those who followed them have only repeated what they had already stated, and were content to approve of a remedy whose uses still lay open to further investigation.

We should then inquire why this medicine has fallen into disuse, as Reil, in his work, asserts that he employed the decoction and extract of the leaves of the holly frequently; and always observed that these preparations caused appetite, facilitated all the secretions and excretions, and that their most constant effect was that of increasing the transpiration of the skin. We also ask, why repeated experiments have not been made with it in cases of intermittent fever, as Reil also affirms that he has employed the holly successfully in epidemic intermittent fever; and he adds, that, after those fevers resisted the use of Peruvian bark, they yielded to that of this indigenous plant. We read in the "Botanique de Lyon," that the dried leaves of the holly, reduced to powder, given in the dose of one drachm in a glass of water, one hour before the paroxysm, frequently cut short the fever.

But all these observations, as well as those of Villars, Murray, &c., seem to have been insufficient to draw from oblivion this medicine, to which I have devoted my especial attention. My inquiries being directed in preference to the febrifuge qualities of the leaves, I omit speaking of the trials made with the berries.

I am now able to present the result of my experiments, which, being verified by enlightened and impartial physicians, demonstrate the great advantages of this medicine as a substitute for Peruvian bark; but, before doing so, it is necessary to consider the best *mode of its administration.*

The leaves of the holly may be administered in different ways: in decoction, in substance, in extract, as bitter principle called ilicine, and in enemata.

1. The *decoction* is prepared by boiling half an ounce of the freshly gathered or dried leaves in eight or ten ounces of water, which is to be boiled down to one half. This quantity is to be given in one dose, two hours before the paroxysm, and to be continued for eight or fifteen days, if the fever does not yield at the fourth or sixth dose.

2. In *substance,* the leaves are to be dried and reduced to powder, and passed through a sieve, and one or two *gros* macerated in ordinary white wine for twelve hours, or made into a decoction with water without being strained. This dose should be given two or three hours before the paroxysm, and, if not found sufficient, the quantity can be increased to three *gros,* which may be repeated four, five, six, or more times, if necessary. Dr. Constantin, of the naval hospital of Rochefort, obtained great success by administering, during the intervals of the paroxysms, one *gros* of the leaves in decoction every four hours, which was continued for two or three weeks, as required, in addition to the vinous prescription.

3. In *extract*, it is given in doses of one half to one *gros*, as a bolus or in the form of pill; or the quantity can be increased to one half, if the pertinacity of the fever should render such increase necessary.

4. As *ilicine*. This febrifuge principle, in which the bitter matter of the plant resides, should be administered in doses of six, twelve, eighteen, or twenty-four grains, in the form of pills, as least disagreeable to the patient; and the use of it continued until the disease is entirely subdued.

5. In *injection*. This mode of administration originated with Dr. Constantin, already mentioned: he recommends half an ounce of the leaves, either dry or freshly gathered, to be boiled for about a quarter of an hour, in a sufficient quantity of water to form a clyster. These injections, besides their febrifuge effects, have the accompanying advantage of producing free evacuations from the bowels, without pain or griping. Dr. Serrurier has likewise prescribed the holly in the form of enemata, and his experience coincides with that of Dr. Constantin, both having been equally successful. Many cases furnished by this physician are embodied in this report. The clinical observations of Professor Magendie have likewise furnished many conclusive facts in favor of the efficacy of this medicine, although they have not been communicated in all the detail that they deserve.

The following cases will give, I hope, a decided proof of the febrifuge action, as well as sedative powers of the leaves of the Ilex aquifolium upon the spleen, liver, and pancreas, especially when the sensibility of these organs has been increased by the use of the cinchona and its compounds; phenomena which, I think, have only been observed by me, and upon which, nevertheless, I am of opinion that the protracted duration of intermittent fever in great part depends.

Case I. John Louis Auscar, aged twenty, coachman, was attacked with a quotidian intermittent fever, the 26th July, 1816; which he said was caused by taking a bottle of beer when he was warm. He entered the Hôtel Dieu, and having left it without receiving any benefit by the treatment he there received, he consulted me the 4th November, 1816, (four months after the first attack of fever.) I ordered him to take one *gros* (in infusion) of the leaves of the holly, two hours before each paroxysm. The second dose cured him, within forty-eight hours. I saw him fifteen days after, and he was quite restored to health.

Case II. A lady, of lymphatic temperament, aged eighteen, afflicted for twelve days with a double quotidian intermittent, in March 1818. She took half a *gros* of the same powder, infused in a glass of white wine, before the paroxysm: one dose sufficed to effect a cure.

Case III. In 1820, Madame Thuillies was afflicted for six months with an intermittent fever: I advised her to try the effect of the holly-leaves, and gave her the powder as above described. Three doses of infusion, each containing one *gros* of the powder, cured her.

Case IV. Madame Cocher, a dairy woman at Ivrey, near Paris, had a quotidian intermittent, for which she was ordered, by a distinguished surgeon, cinchona bark in powder, sulphate of quinine, and wine of Seguin, twice an emetic, &c., without the least relief. She had suffered with this attack for two months and a half before she consulted me: when I saw her, her face was pale, and of a leaden hue; eyes yellow; lips discoloured; tongue yellow; appetite lost; liver enlarged and painful; inferior extremities œdematous; and she could scarcely support herself in the erect position. I ordered her two doses, of two *gros* each, of the same powder infused in wine; the first of which changed the fever to the tertian type, and somewhat restored the appetite. The second dose, which was taken the day after the next paroxysm occurred, produced four alvine evacuations, and entirely cured the fever.

Case V. Joli, aged twenty-one, a labourer at Ivrey, and living in the same district with the milkwoman whose case is detailed above, was treated in a similar way, and by the same surgeon, excepting that he was bled twice; but all without any good effect. Three doses of the powder of holly-leaves, of two *gros* each, were administered. The two first prevented the return of the paroxysms for eight days; and after the third the fever never recurred.

Case VI. M. Despoi, 55, Quai de la Rapée, afflicted with a tertian intermittent during three months, consulted me, June 1827; he had previously taken cinchona bark in powder and the sulphate of quinine. He complained of pain in the epigastrium, which prevented him from walking. The right side was extremely painful to the touch, and I felt, between the liver and stomach, a considerable swelling, deeply seated, which I believed to be a greatly enlarged pancreas; the liver also was more voluminous than natural, and morbidly sensible. Three doses, of two *gros* each, of the holly powder steeped in white wine, restored the patient, so that he had no further relapse of the fever; and the tumor, which I decided to be an enlarged pancreas, gradually disappeared, and the liver lost its preternatural sensibility.

Case VII. Madame M., a lady living at Bondy, was attacked, while walking, in the spring of 1828, with a sudden shivering along the spinal column, which continued for two hours, and was followed by the hot dry stage, and this by the profuse sweating of ague. Similar paroxysms continued to return daily at the same hour for a month, notwithstanding the administration of five grains increased to fifteen grains of sulphate of quinine daily, of two bleedings, and the application of twenty-four leeches to the pit of the stomach. When I was consulted, the 15th June, the face was of a livid yellow colour; eyes yellow; lips blanched; pain in the region of the liver and spleen; and œdema of the limbs. I ordered half a *gros* of the alcoholic extract of the leaves of the holly, to be taken in two doses, three hours before each paroxysm. The first dose caused the paroxysm to continue only one hour, instead of four; the second dose, taken two days after, effected a cure. These two doses produced six evacuations from the bowels.

Case VIII. M. Allard, aged thirty and some years, was attacked, in 1828, with a tertian intermittent, when employed in a porcelain manufactory: he was ordered two grains of tartar emetic, and was bled several times. The fever still continuing, cinchona bark was given in its various forms, which not only created nausea, but irritation of the stomach. The sulphate of quinine was given in boluses, of which he took (according to his own account) 150, four of them morning and evening; did not know the quantity of quinine in each bolus; these effected an intermission of the paroxysms for four, six, and even eight days. At last quinine lost its effect. Hepatitis set in by the continued use of this salt; the least pressure in the region of the liver caused extreme pain, so that he could not endure the weight of the bedclothes, nor even of his shirt. He was advised, at the end of nine months, to leave Champeroux, which was surrounded by marshes: he came to Paris, and consulted me, two months after his arrival, the 25th February, 1829. The fever had then not diminished in the least; there was great weakness; eyes sunk, and slightly yellow; nostrils contracted; lips discoloured; skin generally of a yellow leaden colour; walks with difficulty; loss of appetite; costive and scanty evacuations from bowels; urine of a saffron colour. In this state, I ordered him two *gros* of the powdered leaves, in vinous infusion, to be taken two hours before the paroxysm, which retarded its access from six o'clock A.M. to midday. On the 26th, he had a better appetite than heretofore; but the fever returned on the 27th, at two o'clock P.M. He then took the same dose as before on the 1st March, and the paroxysm did not appear until six o'clock P.M., but was severer than the last. The liver, however, became diminished in size, and he had a greater relish for his meals. The paroxysm returned on the 3d at midnight; also the 5th. Gave him a third dose on the 9th, of two and a half gros of the powder, which completely checked the fever for six days. This dose produced four very fetid alvine evacuations; after which, the patient walked with greater facility, and the irritability of the liver decreased greatly. On the 15th and 18th, paroxysms returned, but were milder. On the 24th, took a fourth dose of three gros. On the 27th, he took the fifth and last dose, which completely subdued the fever. I saw him on the 8th July, perfectly recovered; stomach, liver, skin, &c. performing their functions normally. This was the severest case I have ever seen: I never ordered before such large doses of the Ilex Aquifolium.

I also attended the wife of this person, who had had ninety leeches at three times applied to the epigastrium, and had taken thirty-six boluses of sulphate of quinine without success, for a quotidian intermittent fever. I saw her in 1827, and two doses, of two gros each, of this powder perfectly cured her.

Case X. M. B. was attacked with a double tertian intermittent in February 1829; and after a delay of eight days, in order to remove a rheumatic affection, and to be more certain of my diagnosis, I ordered two doses of the watery extract of the leaves of the holly, each containing one gros, which effected a perfect cure.

Case XI. L. J. Moreau, aged nineteen, living in Paris, of a lymphatic temperament, had a paroxysm of intermittent fever the 24th August, 1829, which lasted twenty hours; also on the 26th, 28th, 31st August, and 2d September; from which time it became a perfect tertian. I ordered him, on the 16th September, two gros of the leaves in decoction, to be taken two hours before the paroxysm. This caused three evacuations from the bowels, and the paroxysm only continued six hours. The fever returned on the 18th; and on the 20th he repeated the same dose, which caused four motions, and the paroxysm continued six hours, but did not return, and he has since enjoyed the most perfect health.

Case XII. I was consulted, the 4th December last, by Madame Voix de Provis, who was affected with a quotidian intermittent for six months. One dose of two gros of the powder, given in wine, effectually removed the fever.

Here terminate the records of those cases which I have myself observed: to the distinguished physicians whose names are mentioned I am indebted for the following, which complete this monograph.

[The author next proceeds to give the details of sixty-five cases of intermittent fever, the greater number of which (forty-three cases) he has collected from the Cliniques of M. Constantin, naval surgeon at Rochefort, and from that of M. Magendie, at the Hôtel Dieu at Paris. The other cases were communicated to him by M. Louis, M. Perronaux, M. Lepredour, &c.

From this very voluminous record, the translator has extracted the most important features, and reduced them to a tabular form, in which the type of fever, the person by whom treated, the medicines administered, and the results are introduced.]

*A Tabular View of Sixty-five Cases of Intermittent Fever, described by* Dr. Rousseau, *in his Prize Essay on the Use of the Ilex Aquifolium in the Treatment of that Disease: with an Appendix, including those Cases treated by the Author himself, and detailed in this Memoir.*

| | Type of Fever. | By whom treated. | Age. | Sex. | Length of time ill. | Duration of Treatment. | Number of Doses, and their Strength and Form. | Page in MS. |
|---|---|---|---|---|---|---|---|---|
| 1 | Quotidian * | M. Constantin | 39 | Male | 6 days | 3 days | 2 doses of 8,00 in wine | 31 |
| 2 | — | — | 20 | — | 8 days | 10 days | 1 dose of 8,00 pulv. | 32 |
| 3 | — | — | 23 | — | 2 days | 14 days | 2 doses of — | 32 |
| 4 | — | — | 23 | — | 2 days | 13 days | 3 doses of — | 33 |
| 5 | — | — | 38 | — | 10 days | 12 days | 2 doses of — | 36 |
| 6 | — | M. Lepredour | | — | — | 3 days | 3 doses of 2 gros in powd. | 48 |
| 7 | — | M. Gillet de S. | 9 | — | — | — | — | 49 |
| 8 | — † | M. Treand | 20 | Male | 12 days | 6 days | 4 doses of 2 gros in powd. | 52 |
| 9 | — ‡ | M. Serrurier | 44 | — | — | — | 3 doses of — | 56 |
| 10 | Tertian | M. Constantin | 23 | Male | 8 days | 4 days | 1 dose of 8,00 of powder | 31 |
| 11 | — § | — | 25 | — | 5 days | 6 days | 3 doses of — | 34 |
| 12 | — | — | 23 | — | 8 days | 8 days | 3 doses of 15 grains, dec. | 38 |
| 13 | — | — | 36 | — | — | 6 days | 3 doses of 8,00 in powder | 39 |
| 14 | — | — | 43 | — | — | 9 days | 1 dose of — | 41 |
| 15 | — | — | 48 | — | — | 5 days | 2 doses of — | 42 |
| 16 | — | — | 52 | — | 10 days | — | 1 dose of — | 42 |
| 17 | — | — | 30 | — | — | 5 days | 3 doses of — | 42 |
| 18 | — | — | 33 | — | — | 8 days | 3 doses of — | 42 |
| 19 | — ‖ | M. Lepredour | | — | — | — | 2 doses of 2 gros in powd. | 43 |
| 20 | — ¶ | M. Serrurier | 49 | Female | — | — | 5 doses of — | 53 |
| 21 | Tert. dou.** | M. Treand | | Male | 7 days | 3 days | 2 doses of — | 52 |
| 22 | Quartan †† | M. Quéau | | Female | 2 mon. | 4 days | 1 dose of — | 30 |
| 23 | — ‡‡ | M. Constantin | 29 | Male | 6 days | 4 days | 2 doses of 8,00 in powder | 33 |
| 24 | — §§ | — | 28 | — | 15 days | 5 days | 2 doses of — | 34 |
| 25 | — ‖‖ | — | 37 | — | 12 — | 13 — | 6 doses of 2 gros in decoc. | 34 |
| 26 | — | — | 26 | — | 15 — | 6 days | 4 doses of — | 35 |
| 27 | — ¶¶ | — | 31 | — | — | 10 — | 3 doses of — | 35 |
| 28 | — | — | 26 | — | 12 — | 12 — | 4 aoses of — | 36 |
| 29 | — *** | — | 32 | — | 10 — | 9 — | 3 doses of — | 36 |
| 30 | — ††† | — | 45 | — | 12 — | 10 — | 6 doses of — | 37 |
| 31 | — ‡‡‡ | — | 31 | — | — | 7 — | 3 injections of 4 gros, p. | 37 |
| 32 | — §§§ | — | 34 | — | 6 — | 10 — | 3 injections of — | 37 |
| 33 | — | — | 23 | — | 10 mon. | 12 — | 3 doses of 15 gr. of powd. | 38 |
| 34 | — | — | 25 | — | — | 13 — | 9 injec. of — | 38 |
| 35 | — | — | 36 | — | — | 12 — | 7 inject. and 2 doses in d. | 39 |
| 36 | — | — | 28 | — | — | 10 — | 3 doses of 8,00 in powder | 40 |
| 37 | — | — | 34 | — | — | 11 — | 4 doses of — | 43 |
| 38 | — | — | 24 | — | — | 11 wks. | 12 doses and 11 injections | 40 |
| 39 | — ‖‖‖ | M. Treand | 69 | Female | 1 year | 7 days | 4 doses of 2 gros in powd. | 51 |
| 40 | — | M. Delonnel | 21 | Male | 24 days | 6 — | 2 doses of — | 67 |
| 41 | — | M. St. Amande | 27 | — | 3 wks. | 8 — | 6 doses of — | 82 |
| 42 | Erratic¶¶¶ | M. Lepredour | 27 | — | 3 — | 6 — | 3 doses of 8 grains, powd. | 45 |

* Convict, as all those treated by M. Constantin.
† Was first bled and purged.
‡ Relapse after six months, cured by quinine.
§ Had frequent attacks of the same.
‖ Relapse: quinine not used, gout being present.
¶ Second attack; first cured by quinine.
** Fifth attack.
†† Quinine failed.
‡‡ Second attack.
§§ Fourth attack.
‖‖ Fourth attack.
¶¶ Second attack.
*** Fourth attack.
††† Relapse.
‡‡‡ Second attack, first cured by quinine.
§§§ Third attack.
‖‖‖ During the last three months, it was a double quartan.
¶¶¶ Quinine failed.

| Type of Fever. | By whom treated. | Age. | Sex. | Length of time ill. | Duration of Treatment. | Number of Doses, and their Strength and Form. | Page in MS. |
|---|---|---|---|---|---|---|---|
| 43 Quotidian | M. Perronaux | 14 | Male | 5 days | 4 days | 4 doses of 1 gros in powd. | 63 |
| 44 — * | M. Cargnou | 47 | — | 14 mon. | 12 — | 4 doses of 3 gros — | 64 |
| 45 — | M. Louis | 29 | — | 1 — | 4 — | 3 doses of 2 gros — | 73 |
| 46 — ‡ | M. Magendie | 25 | Female | 10 days | 25 — | 14 doses and injections | 87 |
| 47 — § | — | 27 | — | 4 — | 4 — | 3 doses of the powder | 92 |
| 48 — | — | 47 | — | 18 — | 12 — | 10 doses of — | 95 |
| 49 — | — | 42 | — | 5 wks. | 4 — | 3 doses of — | 97 |
| 50 — | — | 60 | — | 3 — | 11 — | 9 doses of — | 101 |
| 51 — | — | 38 | — | 3 — | 2 — | 1 dose of decoction | 108 |
| 52 — | — | 62 | — | 11 days | 10 — | 8 d. of decoct. and inject. | 110 |
| 53 — | — | 22 | — | 19 days | 4 — | 3 d. of — — | 113 |
| 54 — | — | 45 | — | 3 wks. | 4 — | 5 doses of decoction | 116 |
| 55 Quot. double | — | 41 | — | 4 — | 12 — | 9 doses of — | 104 |
| 56 Tertian | M. Perronaux | 20 | Male | 10 days | 6 — | 3 d. of 1½ gros in powder | 62 |
| 57 — ‖ | M. Louis | 34 | — | 16 — | 4 — | 2 doses of 2 gros in powd. | 71 |
| 58 — ¶ | M. Arbey | 20 | Female | 6 — | 5 — | 3 doses of — | 78 |
| 59 — ** | M. St. Amande | 18 | Male | 1 year | 3 — | 1 dose of 1 gros in powd. | 83 |
| 60 — | M. Magendie | 19 | Female | 15 days | 15 — | 13 doses of 2 gros and inj. | 85 |
| 61 — †† | — | 23 | — | 14 — | 20 — | 14 doses and injections | 90 |
| 62 — | — | 28 | — | 6 wks. | 9 — | 7 doses of the powder | 114 |
| 63 Tert. dou.‡‡ | M. Collineau | 44 | — | 8 mon. | 5 — | 4 doses of 2 gros in powd. | 68 |
| 64 Intermitt.§§ | M. Serrurier | 76 | Male | 8 days | 20 — | 6 d. of 2 gros and 7 inject. | 57 |
| 65 — | M. Moncourier | 60 | — | 2 mon. | 8 — | 6 doses of — | 75 |

* Quinine, &c. failed. † Left hospital on the fourth day of treatment.
‡ Injections caused copious evacuations.
§ Relapse after seven days, from exposure to cold.
‖ Was previously bled. ¶ Second attack; first cured by quinine.
** Had many attacks, cured by quinine. †† Was exposed to a current of air.
‡‡ Quinine in large doses failed. §§ Injections caused copious evacuations.

### APPENDIX.

*Tabular View of CASES treated by the Author, and detailed in this Memoir.*

| | | | | | | | |
|---|---|---|---|---|---|---|---|
| 66 Quotidian | Dr. Rousseau | 20 | Male | 4 mon. | 2 days | 2 doses of 1 gros in powd. | 17 |
| 67 Doub. quot. | — | 18 | Female | 12 days | — | 1 d. half-gr. pulv. in wine | 18 |
| 68 Intermitt. | — | | Female | 6 mon. | few da. | 3 doses of 1 gros in powd. | 18 |
| 69 Quotidian * | — | | Female | 10 wks. | 3 days | 2 doses of 2 gros — | 19 |
| 70 — † | — | 21 | Male | — | — | 3 doses of — | 20 |
| 71 Tertian ‡ | — | 55 | Male | 3 mon | — | 3 doses of — | 20 |
| 72 Quotidian § | — | | Female | 1 mon. | 2 days | 2 d. one and half gr. extr. | 21 |
| 73 Tertian ‖ | — | 30 | Male | 11 mon. | 6 wks. | 5 d. 2 & 2 and half gr. pul. | 23 |
| 74 Quotidian ¶ | — | | Female | — | — | 2 doses of 2 gros in powd. | 26 |
| 75 Doub. Tert. | — | | Male | 8 days | — | 2 doses of 1 gros extract | 27 |
| 76 Tertian | — | 19 | Male | 3 wks. | 4 days | 2 doses of 2 gros decoction | 28 |
| 77 Quotidian | — | | Female | 6 mon. | — | 1 dose of 2 gros in powd. | 29 |
| 78 Quotid. with Nerv. Fever | — | 11 | Male | 5-6 w. | 16 days | 198 grains of ilicine | 117 |

* Emetics; cinchona and quinine had failed.
† Same treatment and bleeding failed.
‡ Bark and quinine failed: visceral disease.
§ Quinine grs. v. to xv. failed.
‖ Emetics, bark, and quinine failed.
¶ Thirty-six quinine boluses and ninety leeches useless.

### *Summary of the Sixty-five Cases given in detail in the original Manuscript.*

*Twenty-two cases of quotidian intermittent,* principally treated by M. Constantin, of Rochfort, and M. Magendie: equally male and female. One case was fourteen months ill, with whom quinine failed: the fever ceased by the administration of four doses, of three gros each, of the powder of the leaves of the holly, in twelve days. A second case, a female, aged twenty-five, was ten days ill; treatment continued during twenty-five days; fourteen doses, and an equal number of injections of the holly were administered. The remaining twenty cases were ill from two days to five weeks; duration of treatment varied from three to fourteen days, and the number of doses of the holly from one to ten, each of which consisted of from one to three gros of the powdered leaves.

*Twenty cases of tertian intermittent*, fourteen males and six females. One case, treated by M. St. Amande, male, aged eighteen, was one year ill; one dose of the powder, containing one gros, removed the fever. A second case (a double tertian), female, aged forty-four, treated by M. Colleneau; eight months ill; the fever ceased in five days, by four doses, of two gros each, of the powdered holly-leaves. A third case, a female, aged twenty-eight, treated by M. Magendie, was ill six weeks; the fever ceased in nine days, by seven doses of the same powder. The remaining seventeen cases, treated principally by M. Constantin and M. Magendie, were from five to sixteen days ill previous to treatment; duration of treatment varied from three to twenty days, and the doses of the powder from two to fourteen, each containing from one to three gros.

*Twenty cases of quartan intermittent*. One case treated by M. Constantin, male, aged twenty-three, was ten months ill, and was cured in twelve days, by three doses of the powdered leaves. A second case, of a female, aged sixty-nine, treated by M. Triand, was one year ill, during the last three months of which the type of the fever changed to a double quartan, which was cured in seven days, by four doses, of two gros each, of the holly. A third case, of a male, aged twenty-four, treated by M. Constantin; period of previous illness not stated; duration of treatment, ten weeks; nine doses of the decoction of the leaves of the holly were given, each dose consisting of two glasses, the first two weeks; during the third week, three doses of the powder in wine were given. By this treatment the paroxysms became milder, though not less frequent; and during the remaining seven weeks, eleven injections of a decoction of holly leaves (only) were administered, which effectually removed the fever.

The other seventeen cases, sixteen male and one female, principally treated by M. Constantin; periods of illness varied from six days to two months; duration of treatment, from four to thirteen days; doses from one to six of the powdered leaves. In three of those cases, injections of a decoction of the leaves of the holly were only employed, with an equally good effect.

The other three cases of the sixty-five: one of an erratic type, male, aged twenty-seven; three weeks ill; quinine failed, and was cured in six days by three doses of the holly. The type of the second and third cases is not mentioned; males: one, aged seventy-six, was cured in twenty days, by six doses and seven injections of the holly; the other, aged sixty, two months ill, cured by six doses of the holly, in eight days.

*Conspectus of some of the most remarkable Cases, selected from the Sixty-five detailed at length in the original Essay.*

Of the sixty-five cases presented, there are some which merit a more particular notice.

There are four cases given in which quinine, and the other preparations of cinchona bark, failed to produce any change in the fever. The first is that of a female, who during two months was treated for a quartan, by pills of sulphate of quinine and injections of the decoction of bark, without the slightest effect: one dose of the pulvis ilicis, of two gros, was sufficient to check completely the fever. The second in a man, treated during three weeks for an erratic intermittent fever, by cinchona and its preparations, which failed to produce its ordinary effects: three doses of the pulvis ilicis sufficed to effect a cure. The third case, that of a man aged forty-seven, afflicted with a quotidian for fourteen months, during which time he was treated by several persons, some of whom tried the various preparations of cinchona, in the very largest doses: four doses, of three gros each, of the pulvis ilicis effected the long-sought-for cure. The fourth case, that of a female aged forty-four, during eight months was afflicted with a double tertian, for which she took quinine without effect: four doses, of two gros each, of the holly sufficed to effect the cure.

Three other cases deserve notice, owing to the duration of the illness and the comparative smallness of the quantity of the holly necessary to procure a restoration to health. The first, that of a man aged twenty-three, suffering for ten months under a quartan intermittent, which ceased in twelve days after three doses of the ilex. The second that of a female, aged sixty-nine, afflicted during a year with a quartan; four doses of the ilex, of two gros each, effected a cure. The third, a case of tertian intermittent, of one year's standing, which one dose of one gros of the powdered leaves of the ilex effectually removed.

There are six cases (two quotidian, three tertian, one quartan,) cured by one dose, of two or three gros each, of the powder of the holly.

There are ten cases (three quotidian, four tertian, three quartan,) cured by two doses of the same.

There are twenty cases (seven quotidian, seven tertian, six quartan,) cured by three doses of the same.

There are eight cases (three quotidian, one tertian, four quartan,) cured by four doses of the same very valuable medicine.

*Case of Nervous Fever, complicated with daily Paroxysms of Ague, treated successfully by the author of this Memoir, by the use of Ilicine, a substance which he discovered in May* 1831.

Bataille, a youth, aged eleven years, of a nervous habit, residing in Paris for the prosecution of his studies, was entered on the sick list on the 1st of June, 1831. This lad complained of a violent headach, cough, and much heat of the abdomen; but these symptoms were not thought sufficient to confine him to his room, and his medical attendant permitted him to rejoin his class. Being thus neglected, the fever increased in severity, and became manifestly of a typhoid character. On the 27th of June, it was complicated with an attack of ague, which came on about four or five o'clock in the afternoon, the paroxysm lasted two hours and a half. On the 28th the attack returned at eight in the evening; and on the 29th at ten in the forenoon.

I saw the patient at four P.M. in consultation with my colleague Dr. Longes-Villermay. The following were the most prominent symptoms: weakness of the back, much prostration of strength, and general apathy; pupils much dilated, and very little contractile; teeth slightly covered with sordes; mucous rattle in the upper lobes of both lungs; abdomen very hot; urine scanty, and of a red colour; pulse 110 to 120 per minute.

The paroxysms continued to recur daily to the 9th of July, with equal severity, and always commencing with coldness of the feet. Dr. Longes-Villermay proposed the administration of quinine, but I was anxious to employ the febrifuge principle of the holly-leaves, which I had lately discovered; and on Saturday, 9th July, at eight P.M. a pill containing six grains of ilicine was given to the boy, who had a quiet night; he also took at the same time some liquorice powder.

July 10th, at six P.M. The six-grain ilicine pill was repeated; the shivering fit, however, came on, lasting two hours and a half, and the hot stage equalled it in length.

11th. Medicines repeated. The cold stage of the intermittent commenced at two o'clock, and lasted but two hours. The hot stage, however, did not terminate till a quarter to eight in the evening.

12th. Skin more supple, and the patient appears better; but the stupor continues as at first. Ilicine repeated. The paroxysm of fever occurred at eleven A.M., the cold stage lasting for three quarters of an hour: he however seemed less sleepy than on the 11th. At noon he took another six-grain ilicine pill: yet he still had return of coldness in the feet about half-past seven P.M., but it was not followed by sweating.

13th. Two ilicine pills given at six A.M. Coldness of feet came on at half-past nine, and lasted till eleven A.M.; this was followed by intense heat for about an hour, which then gave way to perspiration, and the paroxysm (during which he had a yellow fetid evacuation,) terminated at a quarter-past four P.M.

14th. Medicines repeated. Access of cold stage at half-past ten, lasting only half the time of yesterday's attack, and followed likewise by a hot stage of diminished violence: during his waking intervals he asked for food.

15th. Same medicines. Paroxysm rather longer in cold and hot stages, but terminated by a more profuse perspiration. He slept the greater part of the day.

16th. Took only one pill of ilicine. Paroxysm did not recur till nearly one P.M., and the hot stage was scarcely sensible.

17th. Twelve grains of ilicine given in two pills. Paroxysm delayed till three P.M., and of much less severity.

18th. Twenty-four grains of ilicine given in four pills, beginning at six A.M., and repeating the dose of one pill every three hours. A warm bath likewise ordered.

The same medicines were continued during four days longer, but the paroxysm getting weaker and weaker for two days, and none at all occurring on the 21st or 22d, on the 23d the ilicine was discontinued; and on the 6th of August I saw him for the last time; when he was completely restored to health.

*Note.* One hundred and ninety-eight grains of ilicine were given to this patient, besides which cold lotions were applied to the head, two blisters for a short time to each side of the chest, and to the insides of the thighs: warm fomentations to the abdomen; two warm baths, clysters, pediluvia, and hot cataplasms to the feet. Internally, besides the ilicine, he took only emollient ptisans, gum syrup, capillaire, &c. The above detail shews that this young patient was cured by the use of ilicine, without any recourse being had to bark.

*Conclusion.* The end of our labours, for the present, has arrived; and, assisted as we have been by physicians, the results of whose practice we have already given, we may draw the following conclusions:

1st. That the powder of holly-leaves is, in the cure of disease, a valuable medicine, and that nature, always kind, seems to have designed it especially for that unhappy class of patients which fortune often permits not to consult a physician, and still more frequently prevents from purchasing the medicines he prescribes.

2d. The hospitals, both civil and military, will equally profit by its introduction; and government will find in this febrifuge a great diminution of expense with regard to medicines; as it may be had for the trouble of gathering, and the cost of making those preparations that I have described will be very trifling.

It has been a great gratification to me to have been able to collect so many testimonies as I have cited of its efficacy, and which have been afforded me by the learned physicians whose names have been already mentioned; and it would have increased this gratification could I have added those furnished by Dr. Reynaud, professor and physician at Toulon; Dr. Clement, physician to the Hôpital de la Pitie; Dr. Gaimard, professor and physician at Rochefort; Doctors Serre and Teul, members of the Faculty of Montpellier. But my numerous avocations have prevented me taking notice of the beneficial results which they have obtained, and which have united with others to recommend the holly for trial; and their recommendation has not been made in vain. To all I would return my thanks: they have favored my researches, and as, on the one hand, they have assisted with me to enrich our therapeutic catologues by the addition of a medicine, the properties of which have been discovered again; so, on the other, they have offered to humanity a means, at little cost, to cure those fevers which often yield not at all, or with much difficulty, to the use of bark persevered in for a great length of time; a course so lengthened as often to involve serious disorganization of the liver, independently of the consideration of the high price of the drug, which the poor are unable to afford: a double misfortune this, but one that cannot be objected to the use of the Holly *and its preparations.*

*Note.* Dr. Rousseau's paper was accompanied with specimens of the various preparations of holly in powder, extract, &c., and also with a bottle of the ilicine; all which, together with the original manuscripts, are deposited in the collection of the Medico-Botanical Society, and may be seen on application to the Librarian or Conservator.

